# Non-canonical Functions of Macroautophagy Proteins During Endocytosis by Myeloid Antigen Presenting Cells

**DOI:** 10.3389/fimmu.2018.02765

**Published:** 2018-11-27

**Authors:** Christian Münz

**Affiliations:** Viral Immunobiology, Institute of Experimental Immunology, University of Zurich, Zurich, Switzerland

**Keywords:** LC3-associated phagocytosis (LAP), MHC class I, MHC (HLA) class II proteins, Phagocytosis, autophagy (macroautophagy)

## Abstract

Endocytosis by myeloid antigen presenting cells such as dendritic cells and macrophages regulates both antigen processing and major histocompatibility complex (MHC) molecule trafficking during antigen presentation. The molecular machinery of macroautophagy, a catabolic pathway that delivers cytoplasmic constituents to lysosomal degradation, has recently been found to modulate both MHC class I internalization and phagocytosis of antigens for efficient MHC class II presentation. In this review, I will discuss the respective studies and how these alternative pathways of macroautophagy protein usage differ from their canonical functions. A better understanding of these additional functions of the macroautophagy machinery should allow us to interpret biological effects of macroautophagy protein deficiencies more comprehensively and to therapeutically target the different pathways which utilize the molecular machinery of macroautophagy.

## Introduction

Adaptive immune responses are coordinated and in part executed by T cells. Their activation requires the presentation of non-self peptides on major histocompatibility complex (MHC) molecules (1, 2). These peptides originate from the main proteolytic machineries in cells with the proteasome mainly responsible to produce MHC class I ligands to stimulate CD8^+^ T cells and lysosomal proteases, like cathepsins, predominantly generating MHC class II ligands. These proteolytic machineries do not discriminate between self and non-self, including pathogen derived proteins, but central (e.g., clonal T cell deletion) and peripheral (e.g., regulatory T cells) tolerance mechanisms prevent most T cell activation by complexes of self-peptides plus MHC molecules. Proteasome products reach MHC class I molecules mainly after import into the endoplasmic reticulum (ER) via the transporter associated with antigen processing (TAP), where they are loaded onto co-translationally inserted MHC class I molecules in the MHC class I loading complex containing chaperones and protein disulfide isomerases (1). MHC class I molecules with their octa- to nonameric peptide ligands get then transported to the cell surface for interaction with the T cell receptor (TCR) and CD8 co-receptor of CD8^+^ T cells. The longer MHC class II ligands, often around 15 amino acid long peptides, are primarily generated by lysosomal proteolysis and loaded in late endosomal compartments, that often present as multivesicular bodies (MVBs), called MIICs, onto MHC class II molecules (2). These reach MIICs under the guidance of the invariant chain (Ii) chaperone, which is then degraded by lysosomal proteases and the final Ii peptide remnant is exchanged for high affinity peptide ligands with the help of the HLA-DM chaperone. The resulting complexes of MHC class II molecules and their peptide ligands then gets transported to the cell membrane for interaction with the TCR and CD4 co-receptor of CD4^+^ T cells. According to these cell biological requirements for MHC class I and II ligand generation, CD8^+^ T cells recognize mainly intracellular antigens, and CD4^+^ T cells extracellular antigens after their endocytosis into late endosomes. However, alternative pathways exist for access to proteasomes and lysosomal proteases in MIICs, namely mechanistically poorly defined escape from endosomes during MHC class I cross-presentation, recently coined type 1 cross-presentation, and cytoplasmic constituent delivery to MIICs via autophagy for MHC class II presentation, recently coined type 2 cross-presentation (3).

Autophagy consists of at least three pathways, microautophagy, chaperone-mediated autophagy and macroautophagy (4, 5). So far, only chaperone-mediated and macroautophagy have been implicated in antigen processing for MHC class II presentation (6–9). However, microautophagy also delivers cytoplasmic material to MVBs (10–13) and, thereby, might also contribute to MHC class II presentation of intracellular antigens. While chaperone-mediated autophagy utilizes LAMP2A and cytosolic as well as lysosomal chaperones to transport proteins with a KFERQ-like signal peptide across lysosomal and possibly MVB membranes, macroautophagy employs more than 30 autophagy-related gene (atg) products to build double-membrane surrounded autophagosomes and regulate their fusion with lysosomes and late endosomes, including MVBs (5, 14). The macroautophagy machinery mainly consists of five complexes. The ULK1/Atg1 complex integrates metabolic cues. It is relieved of its inhibition by mTOR and activated via phosphorylation by the AMP-activated protein kinase (AMPK) during starvation. The ULK1/Atg1 complex itself then phosphorylates the VPS34 PI3 kinase complex containing Atg14 on its Beclin-1/Atg6 component, which in turn phosphorylates membranes at which autophagosome formation is initiated, the so called phagophore. Via PI3P the LC3/Atg8 lipidation complex is recruited which consists of Atg5, 12, and 16L1 and conjugates the ubiquitin-like Atg8 proteins, including LC3B/Atg8 and GABARAP/Atg8, to phosphatidylethanolamine (PE) at the phagophore. LC3/Atg8 lipidation then allows for membrane elongation and substrate recruitment, either by directly binding proteins that contain LC3-interacting regions (LIRs) or adaptors that include LIRs and ubiquitin-binding domains to deliver ubiquitinated aggregates, damaged organelles or pathogens into autophagosomes. A fourth complex, that also facilitates endosome maturation, containing VPS34, Beclin-1/Atg6, and UVRAG then regulates fusion with lysosomes, which is executed by the HOPS complex, Rab7 and syntaxin 17 (STX17). While the Atg4 protease recycles LC3/Atg8 proteins from the outer autophagosomal membrane upon vesicle completion, LC3/Atg8 on the inner autophagosomal membrane and its cargo including ubiquitin-binding LIR adaptors like sequestosome/p62 are then degraded by lysosomes. This highly sophisticated machinery of membrane tagging by phosphorylation and then LC3/Atg8 protein conjugation that is used to generate autophagosomes and regulate their fusion with other membrane compartments, is, however, also used for alternative functions including the regulation of endocytosis. This alternative use of the Atg machinery will be discussed in this review.

## Receptor internalization via LC3/ATG8 binding proteins

**Figure 1:**
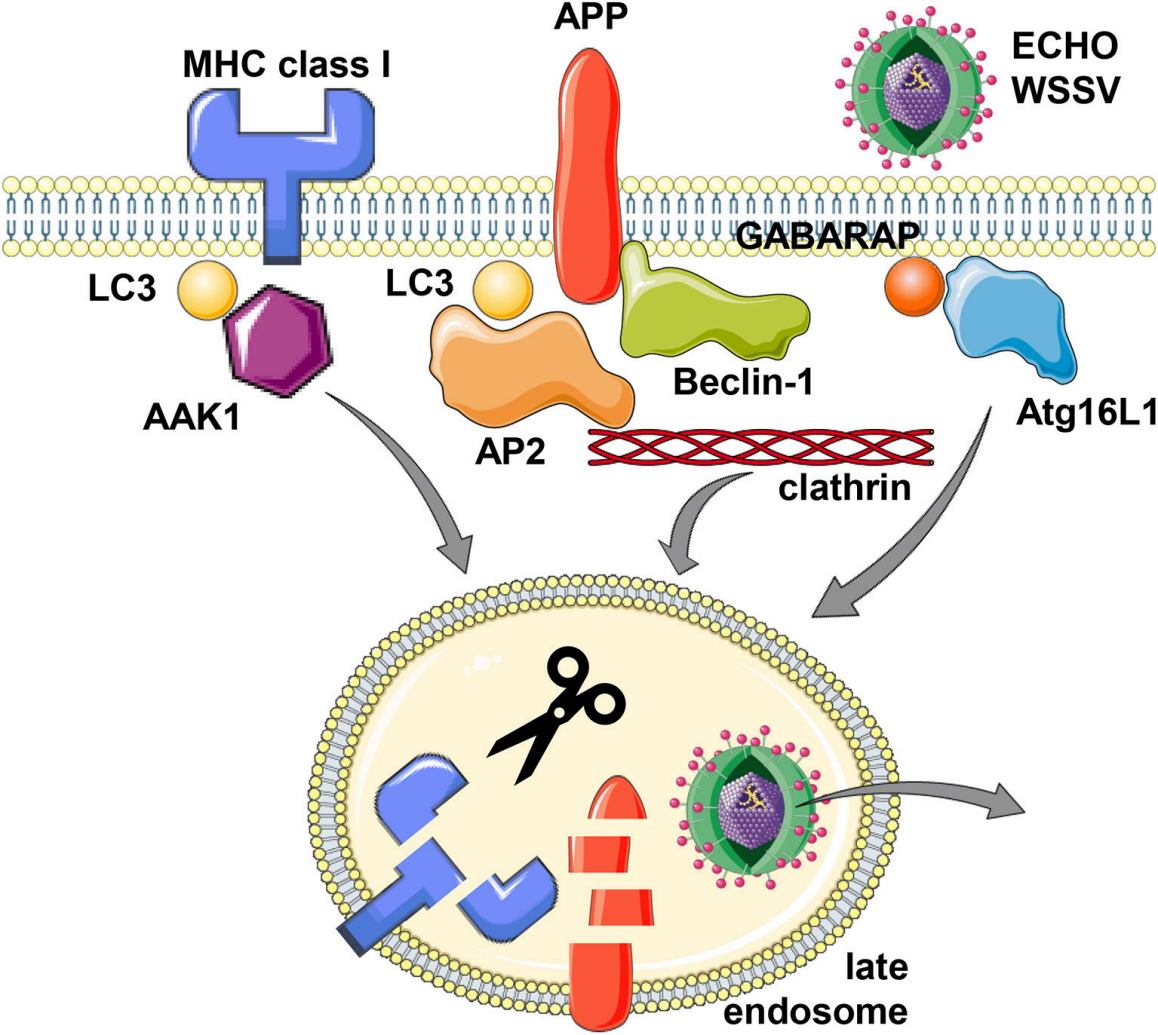
Support of the macroautophagy machinery for receptor and virus internalization. MHC class I molecules, Alzheimer precursor protein (APP) and the two viruses Echovirus 7 (ECHO) and white spot syndrome virus (WSSV) seem to utilize LC3/Atg8 or GABARAP/Atg8 lipidation of the cell membrane for more efficient clathrin dependent internalization. For MHC class I molecules, LC3/Atg8 (LC3) mediated recruitment of the adaptor associated kinase 1 (AAK1), for APP, LC3/Atg8 binding of adaptor protein complex 2 (AP2) and direct binding to Beclin-1/Atg6, and for Echovirus 7 and WSSV, Atg16L1 and GABARAP/Atg8, respectively, have been implicated in their internalization from the cell membrane. While this internalization leads to lysosomal degradation for MHC class I molecules and APP, the two viruses escape to the cytosol from the respective endosomes. This figure was created in part with modified Servier Medical Art templates, which are licensed under a Creative Commons Attribution 3.0 Unported License: https://smart.servier.com.

The first protein for which an involvement of the macroautophagy machinery for its internalization was discovered is the amyloid precursor protein (APP) (15–17). APP proteolysis gives rise to Aβ peptide, the main component of extracellular proteaneous plaques in the central nervous system of patients with Alzheimer's disease (18). It was noted that stimulation of the macroautophagy machinery stimulates APP degradation in a fashion that neurodegenerative Aβ peptides are not produced (15). This protective APP degradation depends on the membrane conjugation machinery of LC3/Atg8 (16) as well as Beclin-1/Atg6 (17). However, lipidated LC3/Atg8 does not recruit APP itself, but rather components of the clathrin dependent internalization machinery, primarily the adaptor protein complex 2 (AP2) (16) (Figure 1). Its AP2A1 subunit contains a LIR motif, which was found to be required for its binding to LC3. Boosting the macroautophagy machinery via starvation or mTOR inhibition increased APP internalization and degradation, and this process was inhibited by RNA silencing of Atg5 (16). In addition to AP2A1, another component of clathrin mediated endocytosis, namely clathrin itself, also contains another LIR motif (19). Furthermore, APP binds directly to Beclin-1/Atg6 via APP's evolutionary conserved domain (ECD) (17). This binding facilitates internalization via the recruitment of the VPS34, Beclin-1/Atg6 and UVRAG containing complex that enhances endosome maturation. Thus, both binding of the clathrin dependent internalization machinery to LC3/Atg8 that is coupled to the cell membrane and Beclin-1/Atg6 binding to APP itself facilitates internalization and degradation of the Aβ precursor and thereby inhibits amyloid generation.

This enhanced internalization with the support of the macroautophagy machinery is also hijacked by viruses (Figure 1). The single stranded RNA virus ECHO (enteric cytopathic human orphan) virus 7 of the picornaviridae family was shown to require core components of macroautophagy for its clathrin dependent internalization (20). RNA silencing of Beclin-1/Atg6, Atg12, Atg14, Atg16, or LC3/Atg8 inhibited echovirus 7 entry prior to uncoating. Of these Atg16L1 was required for echovirus internalization from the cell membrane of intestinal epithelial cells, while attachment was unchanged. Furthermore, the double stranded DNA virus white spot syndrome virus (WSSV) of the Nimaviridae family requires GABARAP/Atg8 for clathrin mediated entry into crayfish cells (21). During entry WSSV colocalized with GABARAP/Atg8 at the cell membrane. Thus, echovirus 7 and WSSV seem to use clathrin mediated endocytosis for surface internalization and this process is facilitated by LC3/Atg8 and GABARAP/Atg8 conjugation to the cell membrane, which might support recruitment of the clathrin mediated endocytosis machinery.

## The role of ATG assisted endocytosis for MHC class I restricted antigen presentation

The internalization mechanism that benefits from Atg8 ortholog mediated recruitment of components of the clathrin mediated endocytosis machinery seems to also influence both classical and non-classical MHC class I molecules (22, 23). In mice that are deficient in the LC3/Atg8 lipidation complex components Atg5 or Atg7 in their dendritic cells and some macrophage populations, these myeloid cells have increased classical MHC class Ia (H2-K^b^ and H2-D^b^) and non-classical MHC class Ib (CD1d) surface expression levels. While these molecules are transported to the cell surface at similar rate in Atg5 or Atg7 deficient cells, their internalization is significantly attenuated. Other surface molecules like MHC class II, CD86 and CD40, as well as MHC class I on B and T cells were not affected by Atg5 or Atg7 deficiency in dendritic cells and some macrophages (22–24). In immunoprecipitation experiments it was found that the adaptor associated kinase 1 (AAK1) interacts with MHC class Ia molecules less efficiently in the absence of Atg5 or Atg7 (22). This kinase phosphorylates the μ subunit of the AP2 complex (AP2M1) for more efficient clathrin mediated endocytosis (25, 26). AAK1 also associates with LC3/Atg8 and contains predicted LIR motifs, suggesting that LC3/Atg8 lipidation at the plasma membrane localizes AAK1 in proximity to MHC class I molecules for their more efficient internalization (Figure 1). Accordingly, RNA silencing of AAK1 stabilizes MHC class Ia molecules on the surface of mouse dendritic cells (22). This MHC class I stabilization is of functional relevance because influenza and lymphocytic choriomeningitis virus (LCMV) specific CD8^+^ T cell responses are more efficiently primed and/or expanded in mice with Atg5 deficiency in their dendritic cells and some macrophage compartments, including alveolar macrophages (22). Influenza infected Atg5 deficient dendritic cells also stimulate virus specific CD8^+^ T cells more efficiently *in vitro* (22). The increased CD8^+^ T cell expansion in mice with Atg5 or Atg7 deficiency in dendritic cells and some macrophage populations also correlates with improved control of viral titers and pathology in the influenza infected mice (22). Similarly, Atg5 or Atg7 deficiency in macrophages rescues mice from influenza induced pathology only after priming of adaptive immune responses (10 days post-infection), while components of the ULK1/Atg1 complex and the VPS34 PI3 kinase complex, Fip200 and Atg14, are also required for the influenza induced pathology during innate immunity at earlier timepoints (27). In addition to this regulation of CD8^+^ T cell responses by altered classical MHC class Ia internalization, NKT cell responses that are restricted by the non-classical MHC class Ib molecule CD1d are also altered in mice with Atg5 deficiency in dendritic cells and some macrophage populations (23). Invariant NKT cells recognize phospholipids on CD1d molecules (28). CD1d accumulation on the surface of Atg5 deficient dendritic cells leads to increased α-galactosylceramide (αGalCer) presentation to NKT cells *in vitro*, and αGalCer injection leads to elevated cytokine production in mice with Atg5 deficiency in dendritic cells and some macrophage populations (23). Furthermore, the pathogen *Sphingomonas paucimobilis*, which is exquisitely sensitive to NKT cell mediated immune control during early infection, reached lower bacterial loads associated with higher cytokine production in the absence of Atg5 in dendritic cells and some macrophages (23). These findings suggest that also NKT cell responses are elevated in the absence of efficient CD1d internalization that is supported by components of the macroautophay machinery.

While endogenous antigen presentation by MHC class I molecules seems to be increased in the absence of LC3/Atg8 lipidation, cross-presentation of exogenous antigens might be compromised (29, 30). Indeed, the pool of MHC class I molecules that is internalized into early endosomes of dendritic cells has been suggested to be required for efficient cross-presentation (31). Dendritic cells with VPS34 deficiency displayed increased LCMV derived antigen presentation on MHC class I molecules to CD8^+^ T cells, but failed to cross-present cell associated ovalbumin efficiently (29, 30). Accordingly, mice with VPS34 deficiency in their dendritic cells and some macrophage populations were more susceptible to challenge with B16 melanoma cells (30). This might suggest that loss of Atg supported MHC class I internalization improves endogenous antigen presentation, but inhibits cross-presentation to CD8^+^ T cells.

## LC3 associated phagocytosis (LAP)

**Figure 2:**
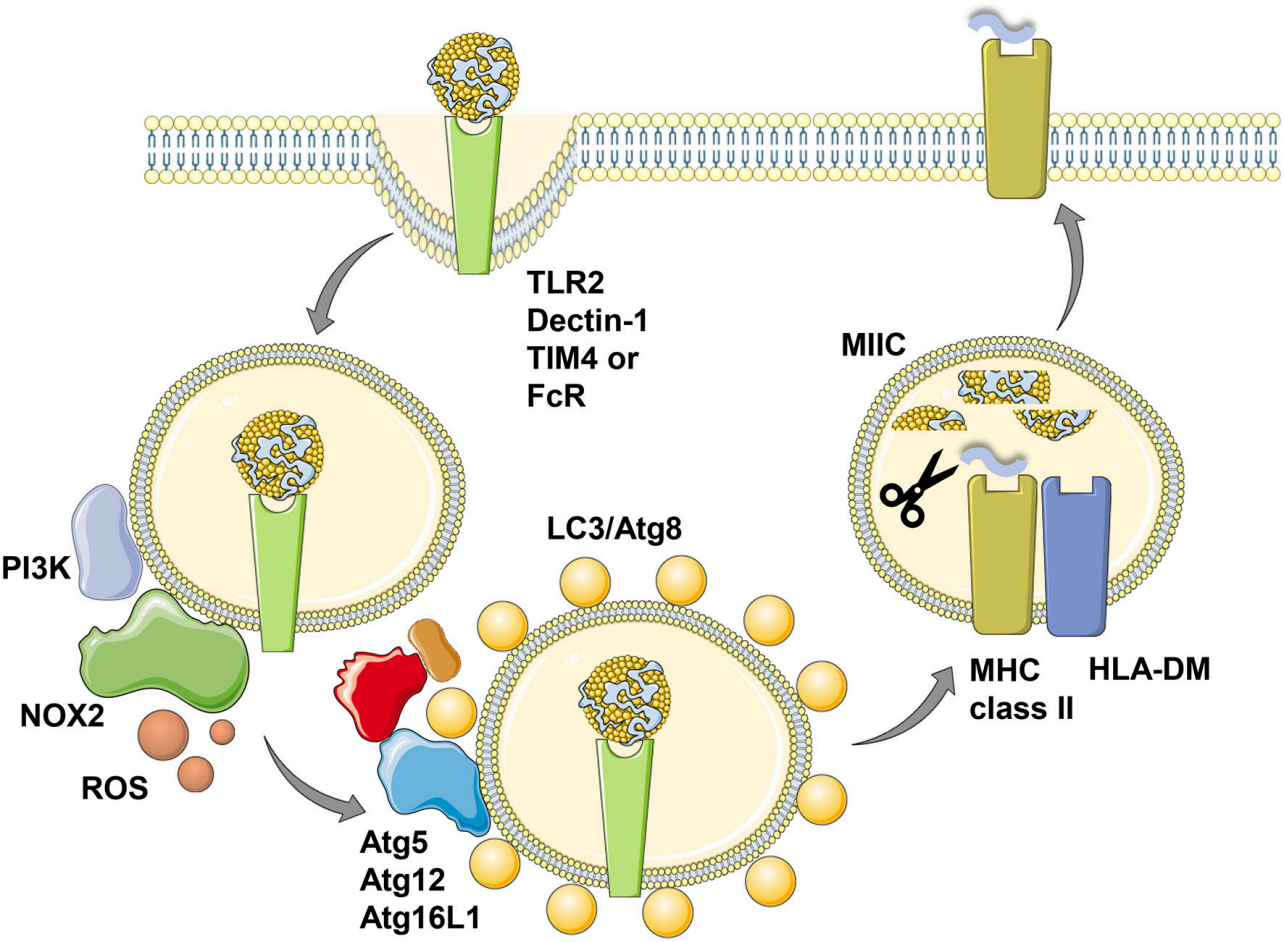
LC3-associated phagocytosis (LAP) of antigens for improved MHC class II presentation. Phagocytosis of ligands for toll-like receptor 2 (TLR2), Dectin-1, T-cell immunoglobulin and mucin domain-containing molecule 4 (TIM4) or antibody Fc receptors (FcR) leads to LC3/Atg8 conjugation to the cytosolic side of the phagosomal membrane in a process called LC3-associated phagocytosis (LAP). Presumably prior to LC3 conjugation this membrane is modified by the PI3 kinase (PI3K) to recruit NADPH oxidase 2 (NOX2), whose reactive oxygen species (ROS) production is required for LAP. The cargo of LC3/Atg8-associated phagosomes is then more efficiently processed for prolonged antigen presentation on MHC class II molecules (MHC class II) which are loaded with lysosomal degradation products in MHC class II containing compartments (MIICs) with the help of the chaperone HLA-DM. This figure was created in part with modified Servier Medical Art templates, which are licensed under a Creative Commons Attribution 3.0 Unported License: https://smart.servier.com.

In addition to LC3/Atg8 lipidation events at the cell membrane that might support receptor as well as virus internalization, such modifications have also been found to take place at endosomal membranes (32, 33). The respective process was coined LC3 associated phagocytosis or LAP (32). It depends on the VPS34 and LC3/Atg8 lipidation complexes, but does not require the ULK1/Atg1 complex (34). This machinery gets engaged when extracellular material is phagocytosed that binds to distinct receptors, including the pathogen associated molecular pattern receptor toll-like receptor 2 (TLR2), antibody Fc receptors, the C-type lectin Dectin-1 and the apoptotic body receptor TIM4 (32, 33, 35, 36) (Figure 2). VPS34 then introduces PI3P marks on the phagosomal membrane, which allow the recruitment of NADPH oxidase 2 (NOX2), whose reactive oxygen species (ROS) generation is required for LAP (33, 34, 37). So far it is unclear how ROS production by NOX2 regulates LAP, but macrophages of chronic granulomatous disease (CGD) patients with defined NOX2 mutations are not able to form LAP phagosomes after TLR2 ligand internalization (33). The LC3/Atg8 lipidation complex can be recruited to phagosomal membranes via the WD40 domain of Atg16L1, which is not present in yeast Atg16 and might be an adaptation in higher eukaryotes to allow for LAP (38). How this WD40 domain, however, recognizes phagosomes that are in need of LC3/Atg8 modification remains unclear. Nevertheless, Atg16L1 mediated recruitment of Atg5 and Atg12 then allows LC3/Atg8 conjugation to the cytosolic side of phagosomes and these membrane tags are only removed prior to phagosome fusion with lysosomes and MIICs (32, 33). LC3/Atg8 conjugation to phagosomes influences their fate differently depending on the cellular background. While it accelerates fusion with lysosomes, possibly by improving endosome transport along microtubules and recruitment of the fusion machinery, in mouse macrophages (39–41), LC3/Atg8 attenuates phagosome maturation and fusion with lysosomes in human macrophages and monocyte-derived dendritic cells (33). In plasmacytoid dendritic cells LC3/Atg8 seems to divert phagosomes to TLR containing endosomes for efficient type I interferon production after phagocytosed pathogen sensing (42). Therefore, LAP seems to regulate endocytosis to adapt the fate of the internalized cargo to the functional needs of the respective phagocyte.

## The role of LAP during MHC class II restricted antigen presentation

Irrespective of the different kinetics of LC3 associated phagosome fusion with lysosomes in human and mouse macrophages, in both species LAP seems to enhance MHC class II restricted antigen presentation (33, 35) (Figure 2). This was shown for *Candida albicans* antigens in human macrophages and for ovalbumin expressed in *Saccharomyces cerevisiae* in mouse macrophages. Accordingly, exogenous antigen presentation on MHC class II molecules to CD4^+^ T cells is also compromised in mice with Atg5 deficiency in dendritic cells and some macrophage populations (24). This also extends to autoantigens, because experimental autoimmune encephalomyelitis (EAE) upon myelin oligodendrocyte glycoprotein (MOG) specific CD4^+^ T cell transfer is severely attenuated in mice with Atg5 deficiency in dendritic cells and some macrophage populations (43). The respective dendritic cells are less efficient in processing apoptotic MOG expressing oligodendrocytes for MHC class II presentation to CD4^+^ T cells also *in vitro*. Commensal specific regulatory CD4^+^ T cells are also less well-induced in mice with Atg16L1 deficiency in dendritic cells and some macrophage compartments (44). Atg16L1 mutations are associated with inflammatory bowel disease (IBD) in humans and patients with such mutations have a decreased frequency of regulatory CD4^+^ T cells. Accordingly, mice with Atg16L1 deficient dendritic cells and some macrophage populations develop inflammatory bowel disease. In this study, the missing regulatory CD4^+^ T cell induction was proposed to be mediated by outer membrane vesicles (OMVs) of commensals. Similarly, mice that lack LAP components in their macrophages were less able to clear apoptotic cells, which led to the induction of autoantibodies resulting in a lupus erythematosus like systemic autoimmunity (45). Interestingly, this phenotype was observed in macrophage deficiencies in Atg5, Atg7, Beclin-1/Atg6 or NOX2, but not affected by loss of the ULK1/Atg1 complex components ULK1 or Fip200. In this experimental system inefficient regulatory CD4^+^ T cell induction could have also contributed to the observed autoimmune phenotype. Therefore, LAP supports anti-fungal, autoimmune and regulatory CD4^+^ T cell responses *in vitro* and *in vivo* by regulating endocytosed antigen processing for MHC class II presentation.

## Conclusions

The above summarized studies suggest that the macroautophagy machinery fulfills important functions for the optimization of endocytosis. So far two stages of endocytosis have been found to be affected by deficiencies in the LC3/Atg8 lipidation machinery, namely early internalization from the membrane, presumably by a more efficient recruitment of the clathrin dependent internalization machinery, and governing phagosome fate by LC3/Atg8 conjugation to the cytosolic side of these vesicles during LAP (46). The ability of the macroautophagy machinery to conjugate LC3/Atg8 to other membranes than autophagosomes has already been realized by Yoshinori Ohsumi, who received for the discovery of the atg genes the Nobel prize for physiology and medicine in 2016. He observed in yeast that was deficient in the Atg4 protease that cleaves LC3/Atg8 from the outer membrane of completed autophagosomes, and that was transgenic for C-terminally truncated Atg8 which is ready for conjugation to PE that LC3/Atg8 could be found on lysosomal, endosomal and ER membranes (47). These findings suggested that LC3/Atg8 deconjugation by Atg4 restricts this membrane tag to autophagosomes in yeast and that any regulatory mechanism of Atg4 mediated deconjugation would allow LC3/Atg8 to be used for other membrane trafficking functions. The identification of such regulatory mechanisms that allows LC3/Atg8 lipidation to be retained at endosome membranes and then used for phagocytosis should clarify in the future how the macroautophagy machinery can fulfill its different tasks during intracellular and extracellular cargo degradation in lysosomes and MIICs.

## Author contributions

The author confirms being the sole contributor of this work and has approved it for publication.

### Conflict of interest statement

The author declares that the research was conducted in the absence of any commercial or financial relationships that could be construed as a potential conflict of interest.
